# People with lived experience (PWLE) of depression: describing and reflecting on an explicit patient engagement process within depression research priority setting in Alberta, Canada

**DOI:** 10.1186/s40900-018-0115-1

**Published:** 2018-10-16

**Authors:** Lorraine J. Breault, Katherine Rittenbach, Kelly Hartle, Robbie Babins-Wagner, Catherine de Beaudrap, Yamile Jasaui, Emily Ardell, Scot E. Purdon, Ashton Michael, Ginger Sullivan, Aakai’naimsskai’piiaakii Sharon Ryder Unger, Lorin Vandall-Walker, Brad Necyk, Kiara Krawec, Elizabeth Manafò, Ping Mason-Lai

**Affiliations:** 1 Department of Psychiatry Faculty of Medicine and Dentistry, University of Alberta, Edmonton, Canada; 2 DRPS Steering Committee, Edmonton, Canada; 3 Patient Engagement Platform, Alberta SPOR SUPPORT Unit, Edmonton, Canada

**Keywords:** Priority setting partnership, Patient engagement, Process, Depression, People with lived experience (PWLE), Depression research

## Abstract

**Plain English summary:**

The Alberta Depression Research Priority Setting Project aimed to meaningfully involve patients, families and clinicians in determining a research agenda aligned to the needs of Albertans who have experienced depression. The project was modeled after a process developed in the UK by the James Lind Alliance and adapted to fit the Alberta, Canada context. This study describes the processes used to ensure the voices of people with lived experience of depression were integrated throughout the project stages. The year long project culminated with a facilitated session to identify the top essential areas of depression research focus. People with lived experience were engaged as part of the project’s Steering Committee, as survey participants and as workshop participants. It is hoped this process will guide future priority setting opportunities and advance depression research in Alberta.

**Abstract:**

**Background**

The Depression Research Priority Setting (DRPS) project has the clear aim of describing the patient engagement process used to identify depression research priorities and to reflect on the successes of this engagement approach, positive impacts and opportunities for improvement. To help support patient-oriented depression research priority setting in Alberta, the Patient Engagement (PE) Platform of the Alberta Strategy for Patient Oriented Research Support for People and Patient-Oriented Research and Trials (SUPPORT) Unit designed, along with the support of their partners in addictions and mental health, an explit process to engage patients in the design and execution of the DRPS.

**Methods**

The UK’s James Lind Alliance (JLA) Priority Setting Partnership (PSP) method was adapted into a six step process to ensure voices of “people with lived experience” (PWLE) with depression were included throughout the project stages. This study uses an explicit and parallel patient engagement process throughout each estage of the PSP designed by the PE Platform. Patient engagement was divided into a five step process: i) Awareness and relationship building; ii) Co-designing and co-developing a shared decision making process; iii) Collaborative communication; iv) Collective sensemaking; and v) Acknowledgement, celebration and recognition. A formative evaluation of the six PE processes was undertaken to explore the success of the parallel patient engagement process.

**Results**

This project was successful in engaging people with lived depression experience as partners in research priority setting, incorporating their voices into the discussions and decisions that led to the top 25 depression research questions.

**Conclusions**

The DRPS project has positively contributed to depression research in Canada by identifying the priorities of Albertans who have experienced depression for depression research. Dissemination activities to promote further knowledge exchange of prioritized research questions, with emphasis on the importance of process in engaging the voices of PWLE of depression are planned.

**Electronic supplementary material:**

The online version of this article (10.1186/s40900-018-0115-1) contains supplementary material, which is available to authorized users.

## Background

Over the last 15 years, engaging people with lived experienced (PWLE) of a health issue, including family and caregivers, in the design, implementation and translation of health research has grown substantially [1–5]. With established and growing recognition of the value of patient engagement in health research activities in the UK [6] and US [7], Canada lagged behind. In August 2011, the Canadian Government launched the Strategy for Patient Oriented Research (SPOR) through the Canadian Institutes of Health Research (CIHR) [8]. SPOR defines patient engagement as “occur[ing] when patients meaningfully and actively collaborate in the governance, priority setting, and conduct of research, as well as in summarizing, distributing, sharing, and applying its resulting knowledge” [9]. The structure to promote the strategy involved the creation of a number of provincial centres called SPOR Support for People and Patient-Oriented Research and Trials (SUPPORT) Units. The Alberta SPOR SUPPORT Unit (AbSPORU) provides the expertise, support, and resources necessary to build and sustain patient engagement in health research throughout the province [10].

A well-established tactic of engaging patients in research is through Priority Setting Partnerships (PSPs). Engaging PWLE in setting research priorities is a structured way of influencing researchers and funders to consider the opinions and needs of PWLE of the specific health conditions. For example, the James Lind Alliance (JLA) developed a template for this type of research in 2004 [11], which has been successfully employed in the United Kingdom (UK), United States (US), and Canada [12–18]. The JLA methodology, among other deliberative engagement tactics, is in response to the history of research topics being selected predominantly by researchers and funding agencies with very little input from patients themselves [19].

In Canada, the prevalence of depression has been relatively stable over time [20]. According to the Canadian Mental Health Association (CMHA), approximately 8% of adults will experience major depression at some time in their lives [21]. Despite the burden of depression in Canada, investment in treatment and research in this area remains low and, consequently, progress on the development of new, more effective treatments is slow. Canada has taken steps to accelerate change to transform Canada’s mental health system, given the direct and indicrect costs are estimated at one-and-a-half times higher than that of all cancers when years of life lost are considered. A specific priority is to build capacity within Canada’s meantal health research community with relevant and timeline research [22].

A recent review article demonstrates a wealth of literature on successful PSPs [23], including nutrition and mental health in Canada [24]. However, a knowledge-to-action gap (K2A) [25] exists not only for depression research in Canada, but also for priority setting partnerships themselves [26]. Despite a growing interest and commitment to including patients in priority setting processes, few articles were identified that reported how patients were meaningfully engaged throughout the priority setting process [27, 28]. Furthermore, methods for engagement and evaluation are often poorly reported or non-existant, limiting opportunities for replication and advancement [23, 29].

To address these knowledge gaps, the purpose of this paper is to describe the patient engagement process used to identify depression research priorities and to reflect on the successes of this engagement approach, positive impacts and opportunities for improvement. This will be described using the GRIPP2 (Guidance for Reporting Involvement of Patients and Public) checklist criteria for patient and public involvement [30] in order to support a consistent approach in reporting process and impact of patient engagement opportunities [31]. It is anticipated that the findings from this study will be specifically meaningful to Albertans as it will reflect the opinions and needs of this population on areas of depression research that they want prioritized. A second paper focusing on the project outcomes is published elsewhere [32].

## Methods

To support this patient-oriented research priority setting in Alberta, the Patient Engagement (PE) Platform of the Alberta SPOR SUPPORT Unit partnered with organizations that have the capacity to reach, advocate for, and represent people with depression, caregivers of people with depression, and clinicians with depression treatment experience. A collaborative partnership was formed with the Addictions and Mental Health (AMH)™ Strategic Clinical Network (SCN™) of Alberta Health Services, and the Alberta Hub of the Canadian Depression Research and Intervention Network (CDRIN) to support a depression research priority setting (DRPS) research project in Alberta with the aim to engage PWLE’s perspectives throughout each stage of the priority setting process. While the predominant role of these partners was to increase awareness of and participation in the initiative among their stakeholder populations in Alberta, the individuals from the partner organizations were involved in the process also and they are working to ensure continual knowledge translation and uptake of the priorities identified. The objective of the Alberta DRPS project was to obtain the input and opinions of patients, caregivers, and clinicians in Alberta on their questions about depression.

The DRPS project was reviewed and received ethical approval by two Research Ethics Boards: the University of Alberta and Athabasca University. The UK’s James Lind Alliance (JLA) Priority Setting Partnership (PSP) method was adapted to gather all questions about depression without domain restriction, including questions beyond the traditional focus of treatment uncertainties, such as “What is the best way to treat psychotic depression in young people?” [33]. The DRPS uses an explicit patient engagement process throughout the entire research processguided by a subject matter experts in patient engagement. Most critically, the patient engagement process was exemplified with the leadership of a patient-led steering committee. There was no JLA advisor guiding the priority setting. Subject matter experts in patient engagement supported the process and the steering committee lead the gathering and collating process of depression research questions into a short list, which were then prioritized into a final list at a face-to-face workshop. The steering committee were objective and task oriented, but prioritized relationship building to prioritize the humanistic versus scientific approach to priority setting. This is reflected throughout the priority setting activities, but critically situated in the initial project planning stage with the formulation of a patient-led steering committee.. The authors felt this adaptation was critical to ensure patients were active decision-making at strategic priority setting tables and had the opportunity to positively influence steering committee discussions and decisions. Furthermore, this increased the likelihood of identifying research questions that were importantant to the patient, thereby eoncompassing more meaningful patient engagement.

**Fig. 1:**
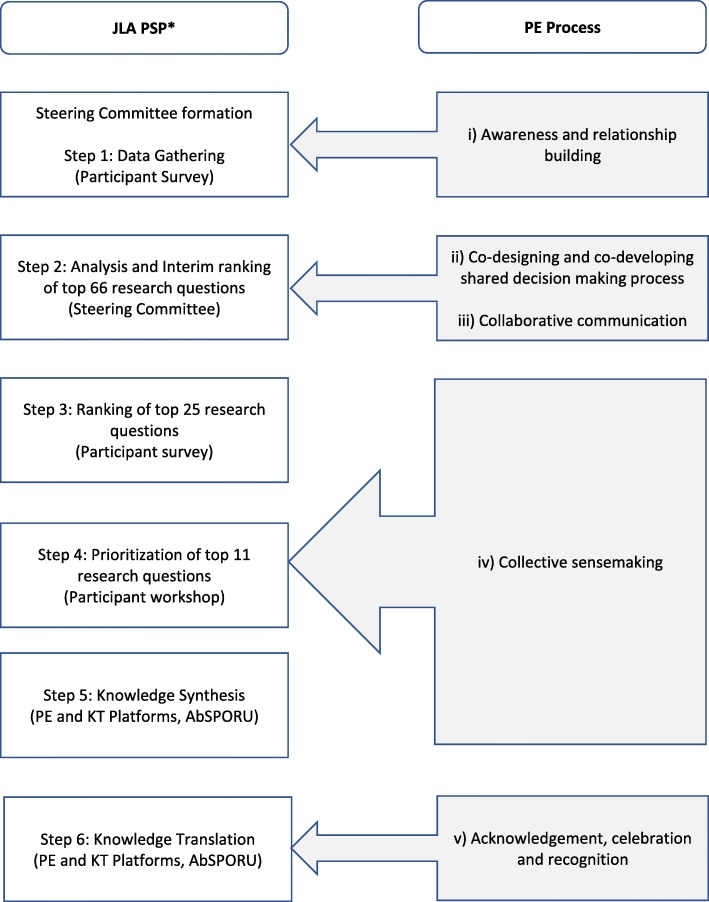
Summary of Depression Priority Setting (DRPS) project and parallel Patient Engagement (PE) process for determining top 25 depression research questions. Legend: * The specific process and outcomes of the JLA PSP method adapted for the DRPS is described elsewhere [manuscript in process]

This demonstration project was led by the PE Platform of AbSPORU to support the participation of PWLE in project co-building and shared decision making. The adapted JLA and parallel PE process is described in Fig. 1. To achieve meaningful engagement, a novel five-step patient engagement approach was developed through reflection and interpretation of the patient engagement process [23, 34]. The following components were confirmed by the Steering Committee and integrated throughout the DRPS’ project process to ensure ethical engagement opportunities for PWLE [35]: i) Awareness and relationship building; ii) Co-designing and co-building a shared decision making process; iii) Collaborative communication; iv) Collective sensemaking and v) Acknowledgement, celebration, and recognition. The DRPS is therefore classified on the ‘Collaborate’ level of patient and researcher engagement in health research, given the equal consensus building process [36] and combination of the perspectives of PWLE and clinicians. The PE Platform, lead by the Program Manager as the patient liaison and subject matter expert, was the point of contact for PWLE. The liaison was key to ensure flexibility and accommodation were provided to PWLE so that their voices were included throughout the DRPS [37]. For example, the patient liaison was considerate of patient schedules and commitments, their capacity to be involved throughout the PSP process, and facilitated the steering committee meeting to ensure the power dynamic between researcher-clinician and patient was well moderated.

### Awareness and relationship building

The identified partner organizations recruited 14 members from their communities to form the project steering committee. This included six PWLE, one caregiver, four clinicians, five researchers and two members of the project planning committee. As evidenced by the total committee numbers, several members represented one or more participant category (e.g., PWLE who is also a researcher). PWLE were recognized for their contributions beyond those of a ‘patient’, rather they were seen as people with different life experiences, both personal and professional (e.g., PWLE identifying themselves as an artist, teacher).

The steering committee was prepared by providing terms of reference,which included the expectations of the term, roles and accountabilities. However, no signing of agreements of partnership were required to build trust and relationship building. Furtermore, no extensive priory involvement was required of members. The steering committee developed the initial data collection survey and collectively analyzed the submitted data across the four-step process on six occasions. To facilitate the parallel patient engagement process, time was taken during every meeting for the steering members to share and connect on a personal level. The first steering committee meeting was face-to-face and included ice breaker activities, which provided opportunities for members to learn about each other. All subsequent meetings commenced with a round table where members would share additional information guided by prompted questions and facilitated by the patient liaison (i.e. wellbeing, celebration of achievements, support from the group, etc.).

### Co-designing and co-developing a shared decision making process

The steering committee collaborated in designing (i.e., planning) the survey for Albertans to provide their concerns and questions about depression. The steering committee met three times to co-develop the survey which was representative of the different perspectives of the steering committee: PWLE, clinicians, and researchers. To facilitate a patient engagement process, PWLE were encouraged to solicit feedback from their communities about the wording, layout, information/data collected, and survey dissemination. This was facilitated directly and indirectly to provide a safe space to provide input by the patient liaison. The title of the survey was created and selected by the steering committee using a consensus building process. All feedback was captured and documented using an online polling system (i.e., Doodle) to arrive at the top titles for decision.

### Collaborative communication

The steering committee worked on communication items, like key messages of the project, and promoted project spokespersons and champions for media outlets. The steering committee remained engaged by different project tasks as the survey was open, while further recruiting participans to provide their input. In-person meetings were prioritized as feasible to foster communication and collaboration. Technological collaboration vehicles were also liberally used including email, tele- and video-conferencing and collaboration tools such as GoogleDocs and Doodle polls for meeting scheduling.

### Collective sensemaking

Data analysis was completed in partnership with PWLE. The steering committee members analyzed the data for diversity of representation to ensure input from many groups was received, including PWLE; carers; clinicians; women; men; seniors and Indigenous populations. This was analyzed periodically throughout the survey (i.e., weekly basis for the first 3 months; every other week for the last month) to inform the steering committee of representation across all responses and allow for outreach to under-represented groups. The proposed research questions were reviewed by the entire steering committee to ensure that they were easy to understand and worded appropriately (i.e. no jargon, acronyms) and reflected the original intent of the questions submitted by Albertans. Questions were also grouped by theme by consensus of the steering committee. In-person meetings facilitated opportunities to ensure the questions were being interpreted using a patient perspective. The aim was to fully capture the nuances in language on the list of prioritized questions. Questions were validated by the steering committee in a face-to-face meeting and then identified to proceed to the literature review process. Differing from the JLA process, a synthesis of the literature occurs earlier to help eliminate research questions that have been addressed by extensive evidence. However, the steering committee felt it important to consider all questions rated by PWLE first before this elimination step to validate their voice throughout the entire priority setting process.

During a full day workshop to prioritize the the research questions, PWLE were paired with clinicians and health care professionals to reinforce mutual understanding of research question selection and prioritization. Participants were divided into three groups to compare their personal rankings. Each group had a facilitator and recorder to manage the dialogue and to guide the process towards group consensus and ranking. Specifically, trained group facilitators who are well -versed and sensitive to the dynamics of the patient-provider relationship, ensured each subgroup had the opportunity to provide input using key principles and understanding of patient engagement. After two iterative rounds of dialogue and small group work, the overall rankings of each question was brought back to the collective group for final decision and ranking. Twenty five questions were included originally, with the group prioritizing 11 questions during the final round for official public release.

### Acknowledgement, celebration and recognition

Best practices in patient engagement literature suggest the importance of providing recognition for patient contributions [2, 38]. People with lived experience were acknowledged in dissemination activities such as conference posters and abstracts and three different PWLE were co-presenters within local and provincial opportunities. Acknowledgement and celebration were not limited to project completion, but also recognized throughout project milestones with ongoing communication and appreciation of contributing efforts (e.g., food, thank you cards, appreciation emails).

Finally, the team recognizes that identifying the depression research priorities is not the most important outcome. Getting answers to the questions is the ultimate goal of all of the partners. The PE Platform is in partnership with other depression group partners and planning organizations to facilitate dissemination and knowledge translation (KT) opportunities that showcase the contribution of people with lived depression experience. As part of this process, PWLE will be acknowledged for their contribution as steering committee members, including as co-author on academic and public publications.

#### Evaluation

The patient engagement literature suggests several considerations when planning for evaluation [39, 40], and strongly advocates for evaluation of the process as a way to promote research learnings [41, 42] as well as further engagement opportunities of translating research findings into practice [38, 42, 43], especially in Canada [44]. Steering committee members were therefore asked to contribute to a formative evaluation of the DRPS project using focus group and key informant interview methodology (*n* = 14 people). The purpose of the evaluation was to explore the value of incorporating the voice of PWLE in research prioroity setting. The two evaluation questioners were: 1) How well did the DRPS gain the perspective of ‘lived experience’ as partners in decision-making at strategic priority setting tables and did this positively influence Steering Committee discussions and decisions?; and 2) How well did the DRPS gain the perspective of ‘lived experience’ as partners in decision-mkig at the “top 10” priority setting tables and did this positively influence the discussions and decisions of the “top 10” collective.The committee evaluated project and process effectiveness by providing feedback on what worked well and considerations for project improvements. The outputs were synthesized into a final report which summarized factors contributing to successful (i.e., meaningful engagement) and challenges that impacted patient engagement opportunities. The report identified recommendations to support future patient engagement efforts.

## Results

This project engaged people with lived depression experience as partners in strategic priority setting and influencing the discussions and decisions of the depression research questions, including the final 25 ranked questions (Additional file 1). It is believed the project accomplished this task because the DRPS was facilitated through strong and continuous participation and commitment of the project steering committee. Three quarters (78.5%, *n* = 11) of steering committee members remained engaged throughout the entire DRPS, while the remaining re-engaged at different time points as permissible.

The authors also feel the project was successful in engaging PWLE meaningfully throughout the DRPS. Even in the final selection of research priorities, 11 questions were selected instead of the intended 10 as a PWLE advocated for inclusion of an additional question because of its reflection of their and so many others experience with depression. Based on evaluation findings [45], steering committee members identified several successes in project effectiveness for meaningful engagement opportunities. These included i) the project’s commitment to in-person meetings and workshops, as feasible, to build trust and mutual respect amongst members and to ensure diverse perspectives were included in the consensus building processes; ii) the flexibility in meeting steering committee members ‘where they are’ within their own journey with depression to participate as feasible; iii) valuing experiences of steering committee members and their networks to father engage stakeholder groups to enhance the reach and impact of this process.

Evaluation data also revealed ideas for improvement. These included: i) Ensuring adequate representation of PWLE when it was not feasible for all members to participate in discussions (i.e., baseline lived experience representation with ratio of 2 PWLE:1 clinician in the event one participant can no longer contribute); and ii) Budgeting for participant compensation to demonstrate recognition and value of steering committee member’s contributions.

Given the success and challenges of engaging patients in health research, it is important to also identify how patient engagement enhances research ouputs. The steering committee identified the perceived positive impacts of engagement with respect to the DRPS project’s contribution to the body of depression research and practice (Table 1), which align closely with what has been captured in the patient engagement literature [31].

**Table 1:** Positive Impacts of Engaging the Voices of People with lived experience

Positive Impacts of Engaging the Voices of people with lived experience	
1. Stories shared by persons with experience of depression (including caregivers) deepen the understanding of others, such as how depression treatments and treatment plans play out in “real life” across individuals, and how this impacts their quality of life. 2. Shared experiences ground priority setting discussions, strategies, and plans in what is important to, and feasible for patients (vs. what is of interest and efficient for clinicians and researchers in new knowledge creation). 3. Perspectives of people with lived experience challenge researcher and clinician assumptions or stereotypes regarding people with lived experience of depression, such as: a. Their depth and breadth of their knowledge (e.g. on the clinical field of depression treatments); b. Their openness to listen to clinical perspectives; c. How their lived experience informs their views on different issues and treatments; d. The value of their contributions and pragmatic suggestions in enriching the quality of priority setting study design and implementation; e. Their willingness and ability to come to consensus with equally willing clinicians and researchers who were open to being informed by their experience. 4. Lived experience perspectives also enrich data interpretation and analysis (e.g. lived experience informs how they would interpret qualitative responses or data provided by other people with lived experience). Sharing this can shift the understanding and interpretations of clinicians and researchers.	

## Discussion

The success of engaging patients is dependent on creating “conditions for success” [18] embedded into project planning and implementation to ensure high and sustained participation from steering committee members.

Patient engagement literature suggests that the sooner patients are engaged, the higher their engagement, which will continue throughout the process since patients are likely to develop their own voices and contribute meaningfully to decision-making processes [38, 43]. The DRPS project first engaged PWLE as members of the steering committee and they remained highly engaged throughout. Success in retention of steering committee members was attributed to several aspects including the time taken to foster face-to-face relationship-building in order to build trust amongst members [46–48], clear leadership from the project planning team [46], and an emphasis on capturing and optimizing the patient perspective across all steps of the DRPS process [41, 48, 49]. This improved the quality of contributions and nuanced information critical to the priority setting process.

Retention of steering committee members with lived experience was also achieved through the purposeful paired discussions between PWLE and clinicians. This not only helped ensure diverse perspectives when analyzing and categorizing the survey data, but fostered mutual respect and support for the patient voice [41, 50, 51]. The DRPS also emphasized the patient voice throughout the ranking and prioritization of depression research questions. As an example, half of the event participants at the final prioritization workshop identified as having lived experience of depression (*n* = 13, 50%; excludes one student guest).

While high participation and engagement in the steering committee was present throughout the project, unforeseeable life events altered participation during the project duration underscoring the importance of flexible engagement [43, 52]. The patient engagement literature highlights considerations for participant flexibility [53, 54] including scheduling meetings well in advance (i.e., over the year’s duration), with consideration to holiday seasons and work flow as well as timing with personal commitments (e.g., end of day). As such, formal and informal mechanisms to engage PWLE were used. For example, PWLE participated through email correspondence, tele- and video conferencing and key informant interviews (i.e., for evaluation). When feasible, a patient liaison was also a key conduit to supporting PWLE, connecting with them about the process and meeting outcomes when they were unable to attend formally scheduled meetings. Furthermore, it is critical that the patient liaison and facilitator of priority setting demonstrate competency and ‘readiness’ to facilitate priority setting activities [55]. Competence in communication, interpersonal and individual relationship building and functioning as a team are critical to successfully engage with persons with lived experience. Furthermore, readiness to deliver can not be underestimated to obtain and sustain interest of PWLE, including ensuring adequate time and resources as well as the maintenance of organizational support, and being prepared to work easily with technology [56–60].

Cost and resource constraints are a key challenge in balancing appropriate and quality patient engagement opportunities with feasibility, particularly in resource restricted environments [43, 61]. It was clearly articulated at the beginning of the project planning that compensation would not be provided to PWLE or clinicians, researchers and health professionals. However, while PWLE were reimbursed for their out of pocket expenses (e.g., parking, mileage, travel and accommodation), compensation for time may better serve in encouraging stronger and sustained engagement by showing the participant that their involvement is valued [62]. At the time, the decision for no compensation was limited by the concurrent development of a policy and procedure document from the national SPOR strategy. This process may have differed with this policy implementation. Addressing operational details to ensure flexibility in the process of engaging PWLE promotes a truly patient-driven study and reduces the possibility of an ‘asymmetrical design’ from the patient perspective where the patient voice is less represented [52].

## Conclusions

Overall, this study contributes to the growing patient engagement enterprise by describing how to effectively and efficiently involve patients in a meaningful, feasible and ethical way, which has been otherwise identified as an ongoing limitation in the patient engagement literature [34]. This study is also a critical opportunity to validate PSP methodologies and frameworks for the most meaningful patient engagement given the relative infancy of this process, especially in Canada. This can include adopting the novel five-step approach of an explicit and parallel patient engagement process which includes a greater emphasis on patient engagement. The illustrative description of both the process and outcomes of the priority setting process used for this study builds on the existing research in Canada [15, 16, 18, 63–69]. This study supports the growth of the evidence on what works in achieving and sustaining productive patient engagement [43].

Dissemination is underway with a public launch of the DRPS to promote further knowledge exchange of prioritized research questions, emphasizing the importance of the process to engage the voices of PWLE with depression. Hopefully, future studies will adapt this process and continue to contribute to patient-oriented research by actively integrating patient engagement throughout priority setting processes.

## Additional file

Additional file 1: Top 11 Depression Research Priorities for Albertans. (DOCX 15 kb)

## Abbreviations

AbSPORU: Alberta SPOR Support for People and Patient-Oriented Research and Trials Unit
AMH: Addictions and Mental Health
CDRIN: Canadian Depression Research and Intervention Network
CIHR: Canadian Institutes of Health Research
DRPS: Depression Priority Setting
GRIPP2: Guidance for Reporting Involvement of Patients and Public
JLA: James Lind Alliance
KT: Knowledge Translation
PEP: Patient Engagement Platform
PSPs: Priority Setting Partnerships
PWLE: People with Lived Experience
SCN: Strategic Clinical Network
SPOR: Strategy for Patient-Oriented Research
