# Supplementary material for: People with lived experience (PWLE) of depression: describing and reflecting on an explicit patient engagement process within depression research priority setting in Alberta, Canada
Source: Res Involv Engagem. 2018 Oct 16;4:37. doi: 10.1186/s40900-018-0115-1 (PMC6190547; doi:10.1186/s40900-018-0115-1)
Supplement: Supplementary file 1 — Top 11 Depression Research Priorities for Albertans. (DOCX 15 kb) [file 40900_2018_115_MOESM1_ESM.docx]

**Additional file 1**

| **Depression priority research questions** | |
| --- | --- |
|  | |
| 1 | Which treatment therapy or method is more successful for long term remission or recovery? |
| 2 | What are the long term physical implications of pharmacotherapy for treating depression? |
| 3 | For various non-pharmacological treatment options (e.g., psychotherapy, individual vs. group psychotherapy, psychosocial support) … |
|  | 1. … what are the advantages in terms of cost? |
|  | 1. …what are the advantages in terms of safety? |
|  | 1. … what are the advantages in terms of effectiveness and relapse prevention? |
| 4 | What are the prevention strategies/tactics for reducing self-harm and suicide in children, youth and adults with depression? |
| 5* | What changes to the health care system will increase access to psychological services? |
| 6* | What changes in the health care system will result in shortened wait times for depression services? |
| 7 | Can diet or exercise affect the development of depression? |
| 8 | What are the functional, social, intellectual, physical and psychological problems experienced by children and teens living with an immediate family member who has depression? |
| 9 | What interventions are effective in preventing and treating workplace depression and reducing stigma associated with depression in the workplace? |
| 10 | Are there structural or functional changes in the brain due to antidepressant therapy during brain development? |
| 11 | What is the role of family in the treatment and trajectory of depression? |
| 12 | Does a mother having depression, or being treated with antidepressants, have measurable effects (positive or negative) on her children? |
| 13 | What is the relationship between the quality of the childhood environment and the likelihood of developing depression in childhood or adulthood? |
| 14 | What are effective public awareness strategies to inform the public about depression? |
| 15 | What is the relationship between hormonal changes and the risk and/or development of depression (e.g. in relation to birth control, post-partum hormonal changes, hormone therapy replacement therapy, and/or menopause in men and women)? |
| 16 | What are the physiological and psychological contributors to depression and how do these change over one’s lifetime? |
| 17 | What kind of additional resources are needed to improve services for people living with depression? |
| 18 | How can schools implement depression prevention programs and address depression in schools to respond to those children and youth who are vulnerable to depression? |
| 19 | How well are health professionals trained in evidence-based best practice guidelines for the diagnosis and treatment of depression at different life stages (e.g. childhood, young adults, seniors)? |
| 20 | How well are health professionals trained in evidence-based best practice guidelines to triage patients with depression in urgent care? |
| 21 | What changes to services are needed to improve outcomes for people living with depression in rural/remote areas? |
| 22 | Are there measurable differences (physical, psychological or neurological) in the growth and development of children with depression? |
| 23 | What are the effects of depression on physical health (such as, but not limited to, migraines, high blood pressure)? |
| 24 | How well are health professionals trained to recognize and treat depression that is associated with another medical condition (such as, but not limited to, anxiety, head injuries, Alzheimer’s, chronic pain, diabetes)? |
| 25 | Does treating depression and addiction at the same time improve health outcomes? |
